# Anatomical Septal Variations in the Maxillary Sinus and Associated Pathological Findings: A Retrospective Cone-Beam Computed Tomography Study

**DOI:** 10.3390/jcm15176791

**Published:** 2026-09-01

**Authors:** Rayan M. Meer, Ahmed M. Kabli, Rawan K. Kamal, Albraa B. Alolayan, Mohamed Omar Elboraey

**Affiliations:** 1 Department of Preventive Dental Sciences, College of Dentistry, Taibah University, Madinah 42353, Saudi Arabia; rmeer@taibahu.edu.sa (R.M.M.); amkabli@taibahu.edu.sa (A.M.K.); rkamal@taibahu.edu.sa (R.K.K.); 2 Department of Oral and Maxillofacial Diagnostic Sciences, College of Dentistry, Taibah University, Madinah 42353, Saudi Arabia; abolayan@taibahu.edu.sa; 3 Oral Diagnosis and Oral Radiology Department, Faculty of Dentistry, Tanta University, Tanta 31511, Egypt

**Keywords:** maxillary sinus septa, sinus volume, maxillary sinus ostium, dental implants, sinus floor augmentation

## Abstract

**Background/Objectives**: Detailed study of the anatomy of the maxillary sinuses is crucial for reducing the risk of complications in sinus augmentation and implant procedures, endodontic surgical treatment, and other operations in the posterior maxilla. This retrospective cone-beam computed tomography (CBCT) study aimed to investigate maxillary sinus volume, septal morphology and distribution, ostium height, and pathological findings, as well as their interrelationships, in a Saudi clinical cohort using combined three-dimensional volumetric and morphometric analyses. **Methods**: A retrospective analysis was performed on 360 CBCT scans obtained from patients after applying predefined inclusion and exclusion criteria. The volumes of the maxillary sinuses were measured by means of semi-automatic segmentation with ITK-SNAP software, while the structure of the septum, anatomical position, orientation, and internal architecture were assessed with BlueSky Bio software. The vertical distance from the natural opening to the bottom of the sinus and pathologies of the Schneiderian membrane were also studied. **Results**: The mean volume of the left maxillary sinus was found to be larger than the right (14,948.6 ± 5473.5 and 13,937.6 ± 4020.7 mm^3^, respectively). No sexual differences were noted with regard to sinus sizes. The prevalence of septa was 80.3% for the left side and 77.5% for the right side. The septa were mostly incomplete, with complete septa being less common. Various types of positioning and orientation were recorded for the septa, with a peak in the molar region. In the case of the left sinus, the height of the ostium was greater in men than women (*p* = 0.007); no differences were observed for the right side. **Conclusions**: The anatomy of the maxillary sinus shows great three-dimensional variation in terms of the shape of septa, the volume of the sinus, the height of the ostium, and any pathological changes related to that anatomy. The significant relationship between the presence of septa and the pathology of the mucosa highlights the need for adequate preoperative CBCT analysis of the anatomy.

## 1. Introduction

The maxillary sinus is the largest of all paranasal sinuses and shows great variability in terms of its shape, size, and pneumatization. Various factors play an important role in causing these variations; some of these factors are craniofacial development, age, sex, erupting teeth, remodelling of alveolar bone, and changes after extraction. Due to the close association between the maxillary sinus and the posterior maxillary teeth, the maxillary sinus has gained much interest in oral and maxillofacial radiology, implant dentistry, endodontics, and oral surgery [1,2,3,4].

Despite the limitations associated with traditional two-dimensional radiographic techniques such as structural superimposition, geometrical distortion, and lack of cross-sectional perspective, cone-beam computed tomography (CBCT) has been found to offer enhanced three-dimensional accuracy with relatively lower radiation exposure; thus, it is the optimal diagnostic method of choice for complex maxillofacial assessments involving anatomical variations of the maxillary sinus [5,6].

Beyond local anatomical variations, maxillary sinus volumes show significant variability between individuals, being largely affected by craniofacial morphology, sex, growth, and development of the skeletal system, as well as maxillary pneumatization [3,4,7]. Despite the availability of 3D volumetric imaging and corresponding measurements, information about maxillary sinus volume and related structural variations among the Saudi population remains limited [8]. Volume analysis becomes necessary in order to be able to predict risk factors of anatomy, minimize surgical complications like oroantral communication, and improve treatment planning in relation to implant surgery, endodontics, periodontal surgery, and maxillofacial surgery [6,8,9].

The presence of septa in the maxillary sinus, is a well-known anatomical variation that disrupts the continuity of the floor of the sinus [10]. These cortical plates segregate the sinus into various recesses. This increases the difficulty involved in elevating the Schneiderian membrane. Meanwhile, asymptomatic inflammatory mucosal pathologies like mucosal thickening of more than 2 mm and mucocele can cause alterations in the ventilation of the sinus because of their interference with the natural ostium [11,12].

Considering the increased use of dental implants as a main modality in today’s clinical setting, along with an increased frequency of performing sinus floor augmentation, especially in cases with advanced alveolar bone atrophy requiring the lateral approach, evaluation of the maxillary sinus septum, any intrasine pathology, and remaining the bone level have become essential for successful treatment planning [13].

Therefore, the present retrospective study aimed to comprehensively evaluate the prevalence, volumetric characteristics, and detailed anatomical distribution of septa, the height of the natural ostium, and pathological findings related to the maxillary sinus in a Saudi dental hospital-based sample, using automated and manual CBCT analysis.

## 2. Materials and Methods

A preliminary retrospective screening was performed on 3974 cone-beam computed tomography (CBCT) records stored at the College of Dentistry, Taibah University, Saudi Arabia. Following a rigorous evaluation based on predetermined selection criteria, a final cohort of 360 high-quality CBCT scans was successfully included in the study.

The inclusion criteria specified scans from individuals aged ≥ 17 years, possessing fully developed bilateral maxillary sinuses, and characterized by superior image clarity devoid of motion or streak/metallic artifacts that could obscure anatomical structures. All evaluated scans had been originally acquired strictly for therapeutic or diagnostic clinical indications [14]. Conversely, scans were excluded if they demonstrated a history of midfacial or maxillary sinus trauma, prior sinonasal surgical interventions, sinus floor elevation procedures, or bone grafting. Scans displaying congenital craniofacial anomalies (e.g., cleft lip or palate), incomplete anatomical coverage of the antral boundaries, or missing demographic/clinical records were likewise excluded [14].

The target sample size was established prior to the study, where the prevalence of the maxillary sinus septa was considered to be the key outcome parameter. Based on a systematic review and meta-analysis, where the patient-level pooled prevalence of maxillary sinus septa was reported as ranging from 28% to 38% [15], an expected prevalence (*p*) of 35% was assumed. The formula for calculating a single proportion estimate is as follows:

$$n=\frac{z^{2}\cdot p\cdot \left(1-p\right)}{d^{2}}$$

where *z* is the standard normal deviate at 95% confidence level (1.96), *p* = 0.35, and d is the maximum acceptable error (0.05). This formula gave the result of 350 scans as the minimum statistical sample size. In order to improve statistical power and include the possibility of non-evaluable scans, the analyzed group included 360 CBCT scans, which exceeded the required minimum sample size. The number of scans analyzed corresponds to other CBCT studies dealing with the maxillary antrum anatomy and variations [15].

This study was conducted in accordance with internationally accepted ethical guidelines for research on human subjects. Patient anonymity was maintained completely before the analysis of data through de-identification of data. The CBCT scans were obtained through the standardization of an operational imaging protocol set by the College of Dentistry of Taibah University, in order to avoid any bias and make sure that measurements were reproducible. Imaging was carried out using a Carestream CBCT unit (Carestream Dental, Rochester, NY, USA), with the same parameters used in all examinations: tube voltage 90 kV, current 4 mA, exposure time 8.01 s, dose area product coefficient 684 mGy·cm^2^, and isotropic voxel size 160 μm. The obtained images were then imported from DICOM format into Blue Sky Bio software (Version 4, BlueSkyBio LLC., Libertyville, IL, USA).

The radiographic analysis was performed retrospectively. In order to avoid the possibility of detection bias, all images were coded and anonymized, stripping away any information that might have identified the patient. The radiographic parameters were independently analyzed by two trained examiners according to a consistent diagnostic scheme. Calibration was performed before starting the process of extracting the data. Additionally, inter-examiner reliability was checked on a random 20% subset of the entire group.

Before the formal assessment, examiner calibration was performed using a pilot set of 15 CBCT examinations that were not included in the final analysis. Interrater and intrarater reliability were assessed in a randomly selected 20% subsample of the CBCT examinations (*n* = 76), which was re-examined after a two-week interval. Intraclass correlation coefficients (ICCs) with 95% confidence intervals (CIs) were calculated for continuous measurements, including sinus volume, mucosal membrane thickness, ostium dimensions, and septal dimensions, using a two-way random-effects model with absolute agreement. Cohen’s κ coefficients with 95% CIs were calculated for categorical variables, including the presence of septa, septal completeness and orientation, and mucosal abnormalities. The ICC values ranged from 0.91 to 0.96 (95% CI: 0.86–0.98), while κ values ranged from 0.85 to 0.92 (95% CI: 0.78–0.96), indicating excellent interrater and intrarater agreement.

### 2.1. Maxillary Sinus Volume Measurement

**Figure 1:**
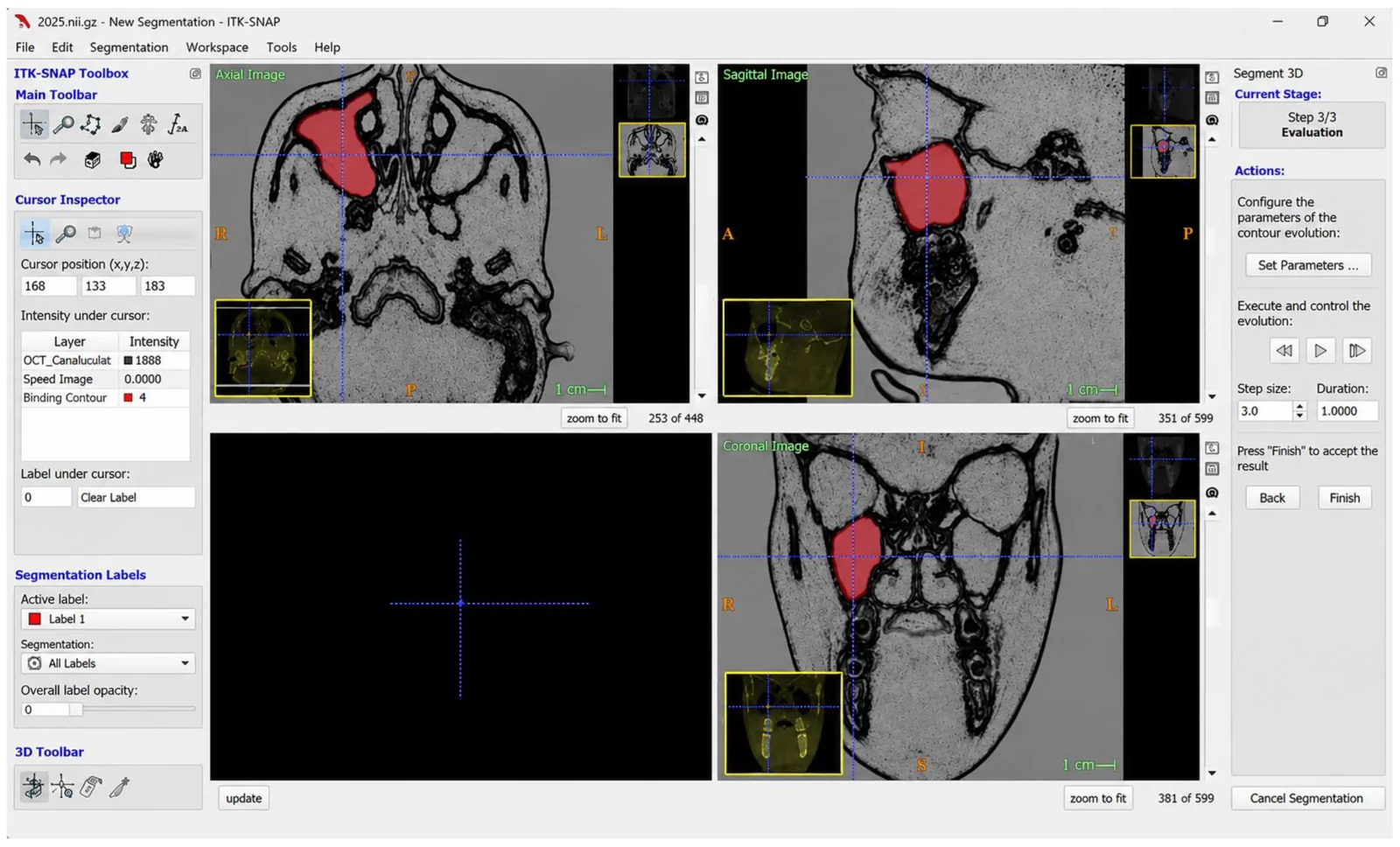
Maxillary sinus volume measurement using semi-automatic, active contour segmentation function according to the snake algorithm using ITK-SNAP software.

**Figure 2:**
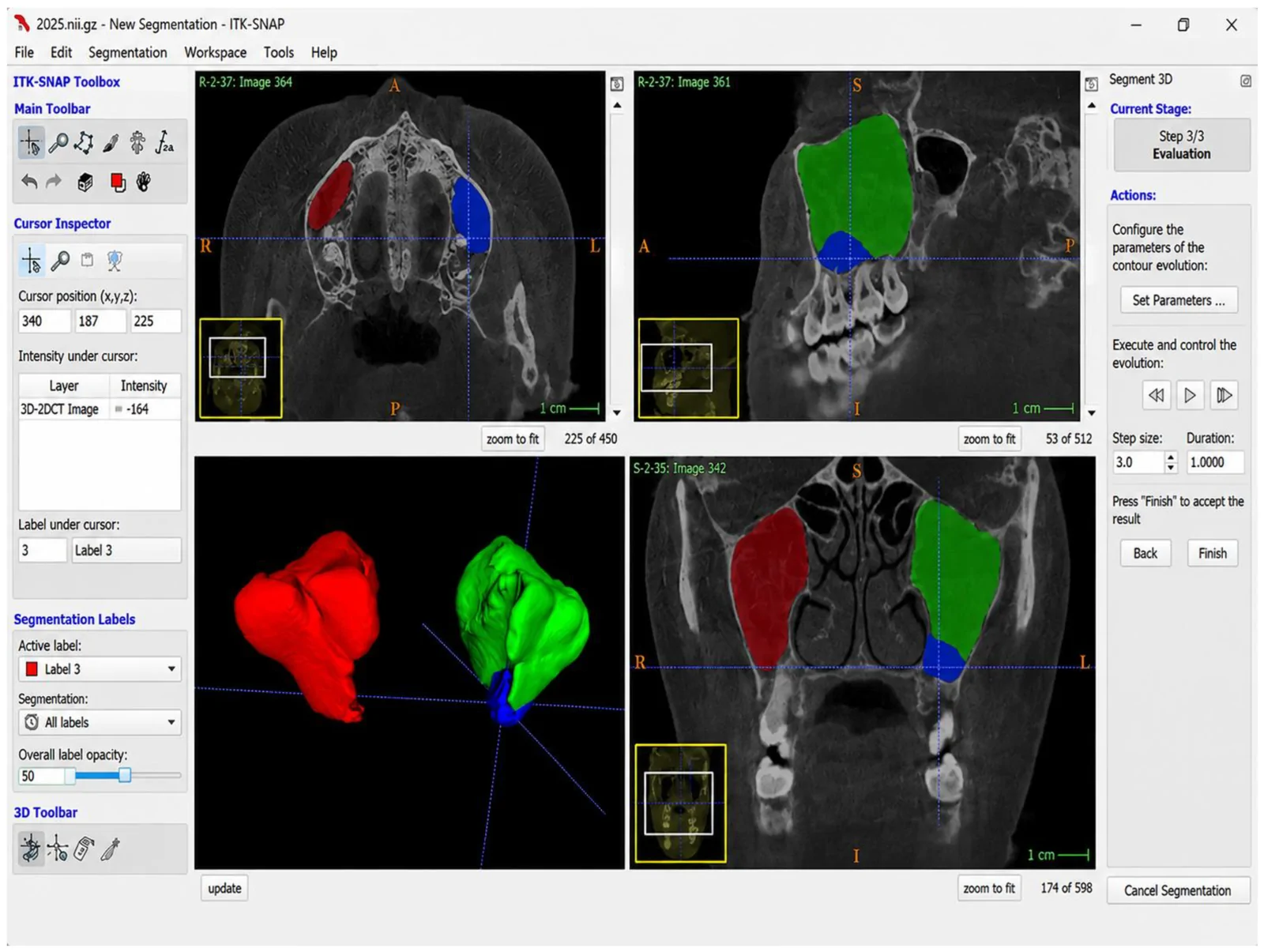
Maxillary sinus 3D volume segmentation by ITK-SNAP software showing right sinus (red), left sinus (green), and sinus pathology (blue).

Maxillary sinus volume was measured using the ITK-SNAP software (Version 3.8.0, Penn Image Computing and Science Laboratory, University of Pennsylvania, Philadelphia, PA, USA). The segmentation process for calculating volume was carried out using the semi-automatic “Active Contour Segmentation” function according to the snake algorithm. For each CBCT image, a specific 3D ROI was created around the target maxillary sinus to segment the maxillary sinus from neighboring anatomical structures. During the pre-processing phase, the “Edge Attraction” option was chosen to guide the movement of the active contours along the highly contrasted bone–air interface of the sinus walls. Following that, the seed points (“bubbles”) with the proper radii were manually positioned inside the antrum, sufficiently far from the sinus cortical margin to avoid the leak. Active contour evolution was subsequently performed, permitting the automatic inflation and fitting of the expanding bubble to the internal spatial structure of the sinus in three dimensions until the entire space is filled. Once the active contour evolution process was complete, the final mesh was generated, and the volume of the maxillary sinus was calculated in cubic millimeters, as shown in Figure 1 and Figure 2 [15].

### 2.2. Maxillary Sinus Septa and Internal Architecture Protocol

Extensive qualitative and quantitative evaluations of the internal antral structures, particularly the maxillary sinus septa, were carried out using Blue Sky Bio software (Version 4, BlueSkyBio LLC., Libertyville, IL, USA). To facilitate the visualization of the internal sinus cavity without obstruction, a “de-roofing and lateral wall resection” approach, which involved two directions of evaluation, was employed on the constructed 3D models.

Initially, to enable the volumetric assessment of the antral floor and internal walls from superior to inferior, a de-roofing approach, where the superior sinus wall (the orbital floor) was virtually resected, was used. Following this, to ensure accurate diagnosis and reduce errors caused by the anatomical shadows, lateral wall resection (lateral window protocol) was performed to provide the second viewpoint to evaluate the internal structure.

**Figure 3:**
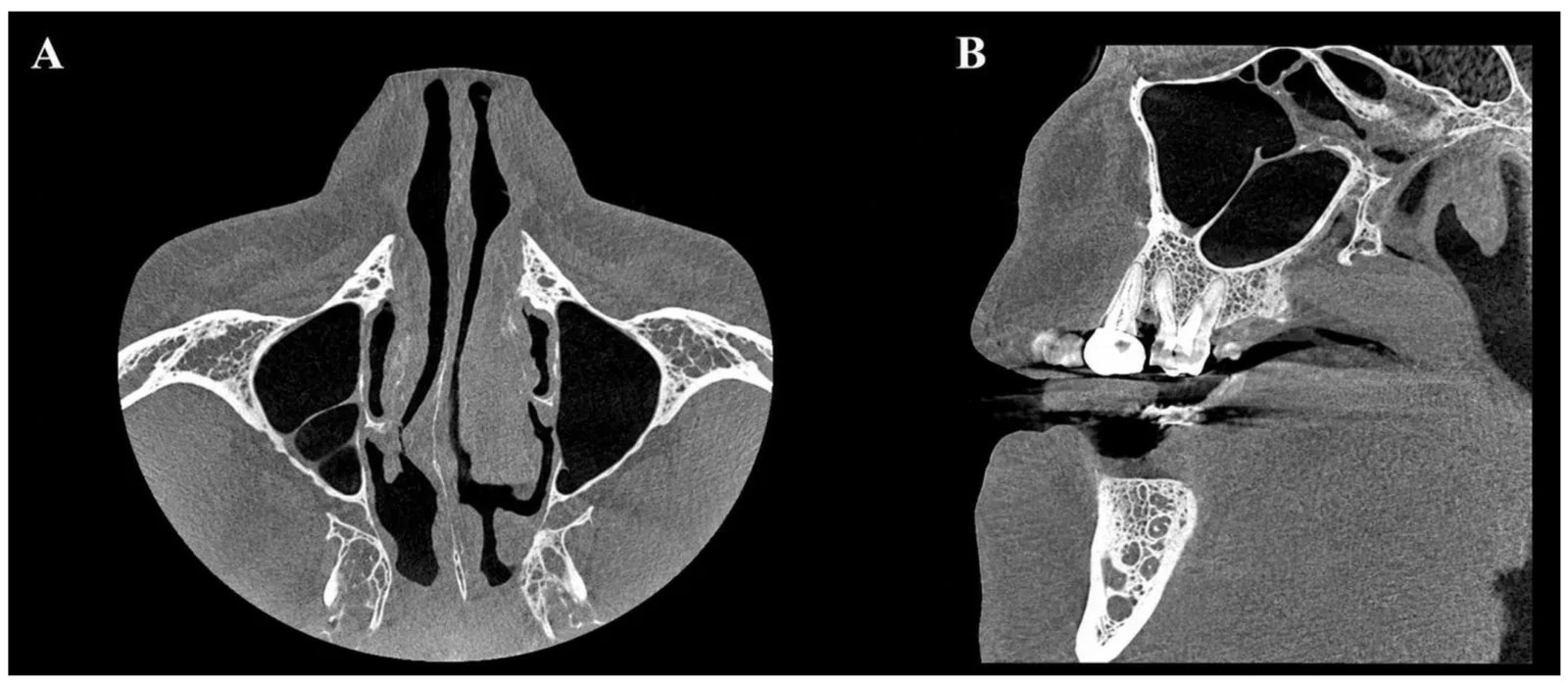
Maxillary sinus septal interpretation by BlueSky Bio software (**A**) showing axial view, (**B**) showing cross-sectional view.

**Figure 4:**
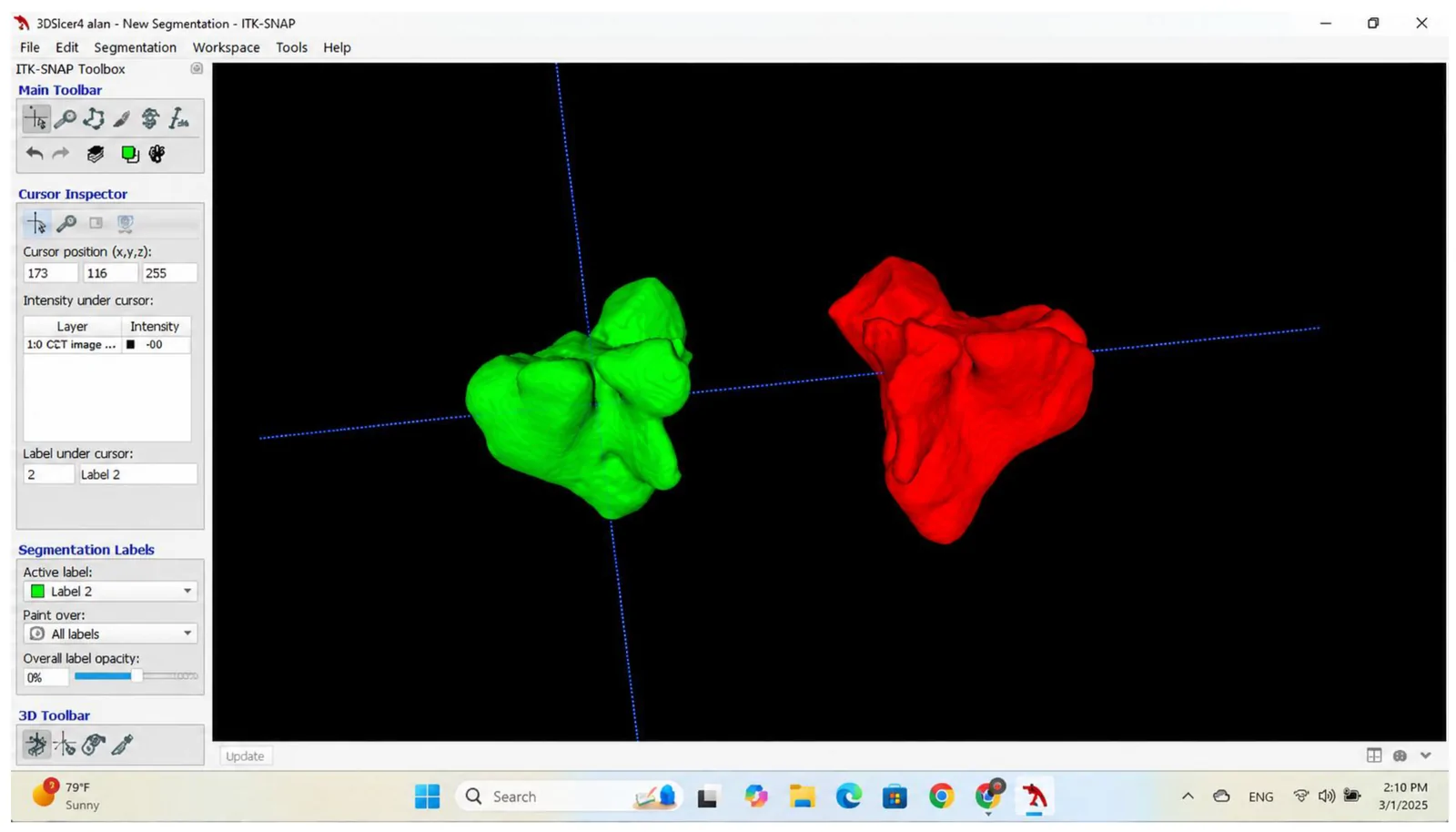
Maxillary sinus 3D segmentation showing grooving by maxillary sinus septa.

For each identified septum, the following comprehensive parameters were recorded, as shown in Figure 3 and Figure 4:

1. **Prevalence and Frequency:** Presence/absence and the exact number of septa per sinus (solitary vs. multiple).
2. **Completeness (Structural Extent):** Categorized as **Complete** (dividing the sinus cavity entirely into distinct isolated compartments) or **Incomplete** (partial bony projections arising from the sinus walls or floor without fully partitioning the cavity).
3. **Cavity Compartmentalization:** Assessment of the resulting internal architecture, recording the precise number of distinct intra-sinus compartments formed by the septal arrangements.
4. **Location:** Mapped according to its anterior–posterior position along the sinus floor into **Anterior** (premolar region), **Middle** (first/second molar region), or **Posterior** (third molar/tuberosity region).
5. **Orientation and Direction:** Classified according to its primary spatial course into anterior posterior, mediolateral, and/or superior inferior.

### 2.3. Measurement of Distance from Maxillary Sinus Ostium to the Sinus Floor

The distance between the lower border of the ostium of the maxillary sinus and its floor was measured based on coronal CBCT sections analyzed using ITK-SNAP software, Version 3.8.0. 

### 2.4. Pathological Screening and Schneiderian Membrane Evaluation Protocol

Comprehensive screening for maxillary sinus pathologies and mucosal abnormalities was conducted using a systematically standardized multiplanar and three-dimensional (3D) approach. Each CBCT volume was thoroughly inspected across all orthogonal planes—axial, coronal, and sagittal views—and further cross-verified on the rendered 3D volumetric model following the digital de-roofing procedure.

In order to determine the presence and nature of mucosal pathology, the thickness of the Schneiderian membrane was determined vertically with respect to the underlying bony floor and walls, up to the point where the membrane was most elevated. In accordance with the already set standards of radiographic diagnosis of clinical conditions, a thickness of ≤2.0 mm was considered as normal mucosa, and thickness greater than 2.0 mm as mucosal pathology [11,12].

**Figure 5:**
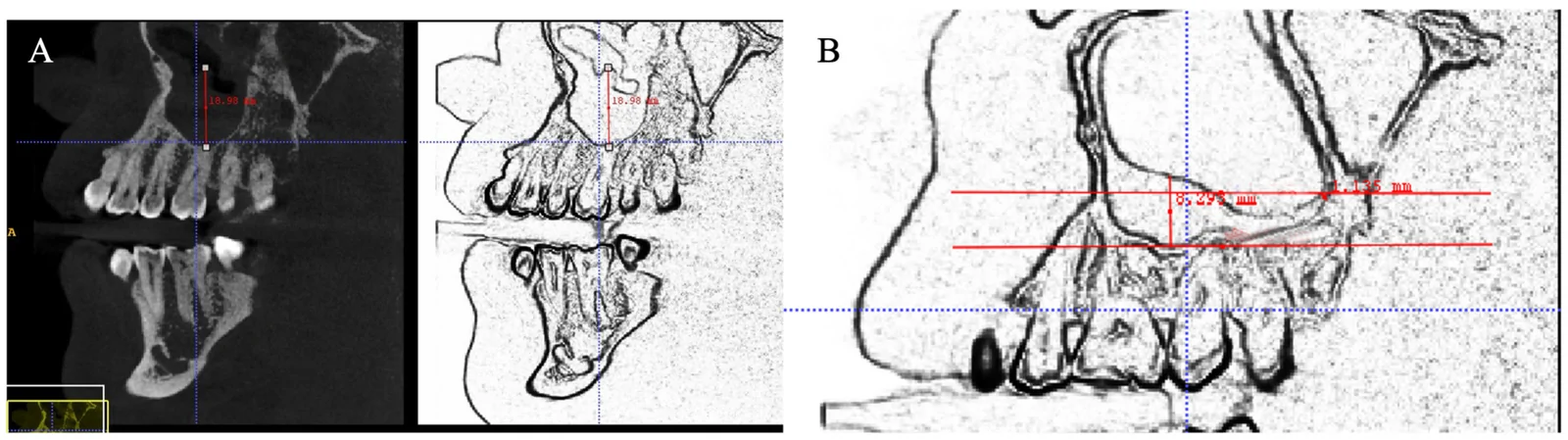
Coronal CBCT views of maxillary sinus demonstrating Schneiderian membrane thickening. (**A**) Extensive Schneiderian membrane thickening occupying nearly the entire maxillary sinus cavity. (**B**) Localized Schneiderian membrane thickening.

For each case of identified pathology, the following clinical factors were determined, as shown in Figure 5:

1. **Prevalence and Classification of Pathology:** Identification of specific pathological entities, including generalized/localized mucosal thickening (>2.0 mm), polypoid lesions, retention cysts, mucoceles, and partial or complete sinus opacification [11,12].
2. **Maximum Membrane Thickness:** Quantitative measurement of the peak vertical height of the mucosal thickening in millimeters (mm).
3. **Anatomical Localization:** Detailed mapping of the pathological manifestation within the antral cavity (categorized into floor, anterior, posterior, medial, lateral, or apical region relative to adjacent root tips).

### 2.5. Statistical Analysis

Statistical analysis was performed with the SPSS software package (Version 29.0, IBM Corp., Armonk, NY, USA). Assessment of normality of distribution was conducted via the Shapiro–Wilk test and visual inspection of histograms. The statistical unit for the analysis of demographics and comparisons of independent subject groups (sex differences, for example) was a single patient. Sinus-specific parameters were tested taking into consideration individual sinus as the statistical unit of study, and intrapatient correlation for the left–right side comparison of bilateral measurements.

Normally distributed quantitative variables are presented as mean ± standard deviation (SD) and were analyzed by comparing groups via Student’s *t*-test for independent samples. Nonparametric quantitative variables are shown as median (IQR) and were tested with the Mann–Whitney U test for independent samples, and the Wilcoxon test for paired bilateral comparisons. Qualitative parameters are given as frequency (percent). Statistical analysis of categorical variables was carried out by Chi-square test or Fisher’s exact test; paired categorical data (left vs. right) were tested with McNemar’s test. A two-sided *p*-value < 0.05 is statistically significant, and exact *p*-values are reported together with respective results.

## 3. Results

This study involved 360 cases evaluated via cone-beam computed tomography. The population age was between 17 and 61 years old, with a mean age of 33.5 ± 12.89 years. An equal sex composition in terms of males and females (50%) was evident in the studied population.

### 3.1. Maxillary Sinus Volume

In regard to the left maxillary sinus, the values of the volume varied between 4903 and 28,170 mm^3^, with a mean value of 14,948.6 ± 5473.49 mm^3^. The right maxillary sinus volume was 6842 to 20,150 cubic millimeters, with a mean and standard deviation of 13,937.6 ± 4020.68 mm^3^.

Comparative analysis of maxillary sinus volume between males and females revealed statistically significant differences on the left side. The mean volume was 16,103.93 ± 5644.36 mm^3^ in males and 13,793.33 ± 5299.66 mm^3^ in females, with a *p*-value of 0.001. On the right side, the mean volume was 13,885.6 ± 4431.11 mm^3^ for males and 13,989.76 ± 3721.01 mm^3^ for females, yielding a *p*-value of 0.81.

### 3.2. Septal Variation Analysis

Regarding the prevalence of maxillary septa, analysis of the right sinus demonstrated the presence of septa in 279 cases, representing 77.5% of the sample. On the left side, the prevalence differed between sexes, with 289 cases, representing 80.27% of the sample.

### 3.3. Septal Location

Regarding the anatomical distribution of mediolateral direction of septa on the floor of the left sinus, maxillary septa were observed across the midline of tooth #24 in 8.57% of cases, at tooth #25 in 2.86%, at tooth #26 in 31.43%, at tooth #27 in 11.43%, and at tooth #28 in 8.57%. The remaining 37.14% of septa were distributed across the lateral regions of the sinus floor, positioned interdentally, laterally to the roots, or slightly anterior/posterior to the designated tooth sites.

Regarding the anatomical distribution of maxillary septa on the right side, maxillary septa were observed at tooth #14 in 6.67% of cases, at tooth #15 in 3.33%, at tooth #16 in 23.33%, at tooth #17 in 33.33%, and at tooth #18 in 3%. The remaining 30.34% of septa were located across the lateral sinus floor, extenting interdentally, laterally relative to the dental roots, or slightly beyond the designated tooth positions.

### 3.4. Septal Extent

**Figure 6:**
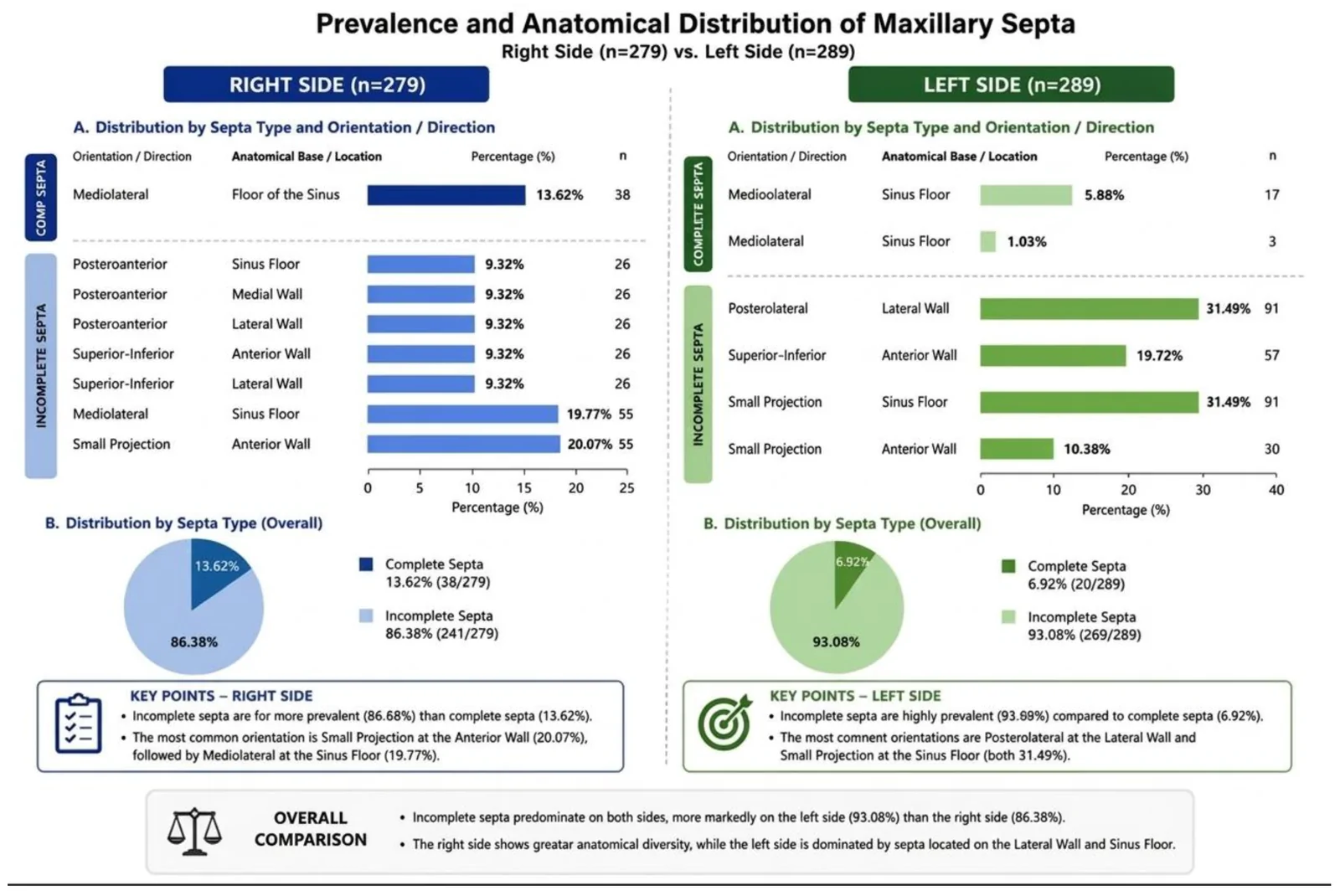
Prevalence of anatomical distribution of maxillary septa.

As detailed in Table 1, analysis of right-sided maxillary sinus septa (*n* = 279) revealed a predominant occurrence of incomplete septa over complete forms (13.62%, *n* = 38), with all complete septa consistently demonstrating a mediolateral orientation restricted to the sinus floor. Conversely, incomplete septa showed a great variety of morphological characteristics regarding their directional orientation and basilar placement. The configuration posteroanterior was equally presented on the floor, medial, and lateral wall (9.32% each), while superior–inferior direction was exclusively localized in the anterior and lateral wall (9.32% each). It is noteworthy that the highest occurrence in terms of incomplete septa was observed in small septal projections at the anterior wall (20.07%, *n* = 56), while mediolaterally oriented septa occurred on the floor of the sinus (19.71%, *n* = 55), as shown in Figure 6.

Regarding the left maxillary sinus (*n* = 289), as shown in Table 2, incomplete septa dominated over complete septa; the later constituted only 6.91% (*n* = 20) cases, including posteriorly lateral (5.88%, *n* = 17) and mediolateral (1.03%, *n* = 3) oriented septa, which were found exclusively in the floor of the sinus. On the other hand, incomplete septa had greater structural variety, with the most common ones being posteriorly lateral on the lateral wall and small projections on the floor (each 31.48%, *n* = 91). Additionally, anterior superior–inferior orientations situated on the anterior wall comprised 19.72% (*n* = 57) of the findings, while small projections on the same wall represented the remaining 10.38% (*n* = 30) as shown in Figure 6.

### 3.5. Ostum Height

Regarding the vertical height of the ostium, a significant difference was observed between males and females on the left side, with males showing a greater height, whereas the difference between the sexes was not statistically significant on the right (Table 3).

### 3.6. Radiographic Pathological Screening of Maxillary Sinus

Analysis of the maxillary sinuses revealed distinct radiographical pathological patterns bilaterally. In the left maxillary sinus, mucosal thickening was observed in 101 patients, while mucoceles were identified in 63 cases. In comparison, the right maxillary sinus demonstrated mucosal thickening in 63 patients, including 9 patients with mucosal thickening of the roof of the sinus and another 9 cases with mucosal thickening that almost completely obstructed the sinus cavity. Mucoceles in the right maxillary sinus were observed in 63 patients (Table 4).

### 3.7. Correlation Between Maxillary Sinus Septa and Pathological Findings

A statistically significant relationship was established between maxillary sinus septae and pathological changes in mucosa (*p* < 0.001, *χ*^2^ test). In particular, of the 720 studied sinuses, there was a significantly higher frequency of thickened membrane (Schneiderian membrane > 2.0 mm) (148 sinus, 26.1%) in sinuses with septae (*n* = 568) in comparison with sinuses without septae (16 sinus, 10.5%), OR = 3.00 (95% CI: 1.73–5.20, *p* = 0.0001). Regarding mucosal thickness, the difference between these two groups (Welch *t*-test) was statistically significant, as septated sinuses had a significantly higher mucosal thickness (3.82 ± 1.14 mm) compared to non-septated sinuses (1.65 ± 0.42 mm), with a mean difference equal to 2.17 mm (95% CI: 2.05–2.29 mm, *p* < 0.001). Moreover, complete septa were found to be more often associated with mucosal opacity and retention cysts than incomplete septa, an association that was detected in 112 cases (19.7%) of septated sinuses vs. 14 cases (9.2%) of non-septated ones (OR = 2.4).

## 4. Discussion

Anatomical complexity and pathological condition of the maxillary sinus are major factors that influence the predictability and safety of more complex maxillofacial surgery procedures, such as sinus floor elevation, dental implants, and endodontics in the posterior maxillary region. The current retrospective study using CBCT technology describes in detail the mapping of volume of maxillary sinus, septa prevalence, natural ostium height, and mucosal pathologies in a Saudi dental hospital-based sample. Through 3D segmentation and visualization techniques, this study provides significant anatomical data relevant for pre-surgical risk estimation and treatment planning. 

A clinically impactful finding of this study is the significant correlation between septation of the sinuses and pathological changes in the mucosa (*p* < 0.05). Functionally, septa provide a barrier that divides the main antral cavity into secondary spaces. Consequently, mucociliary clearance of the sinus is hindered, leading to mucus retention in specific areas, poor gas exchange, and hypoxia of tissues [16].

This altered physiological microenvironment causes bacterial stasis and chronic subclinical inflammation leading to Schneiderian membrane thickening, cysts, or mucoceles. Furthermore, septa in the middle sinus (molar region) demonstrated the most pronounced mucosa thickening (>2.0 mm). From a clinical point of view, the coexistence of structural bone septum with an inflamed and friable Schneiderian membrane is a high-risk surgical environment. During lateral window sinus augmentation procedures, the lifting of such membrane over the septal crest is associated with a higher risk of membrane perforation with the subsequent exposure of the bone graft to infection and possible early implant failure [11,12,17,18].

The morphology-based classification described in this study is in agreement with previously known anatomical classifications, which distinguish between the primary and secondary septa based on developmental differences, as explained by Underwood (1910) [19], where primary septa develop during the embryonic development of the tooth while secondary septa develop due to the unequal resorption of the alveolar process following tooth extraction. The more frequent occurrence of septa in the central part (molar area), with these being mostly transverse/sagittal in orientation, is consistent with the complicated stress patterns in the posterior maxilla during mastication.

A proper classification of septa, with respect to their exact orientation and structure (complete and incomplete septa), is crucial for surgical preparation. Complete septa dividing the antrum into multiple sub cavities create more surgical difficulties compared to incomplete ridges. Complete septa are more likely to result in tears during standard membrane reflection procedures, because of the unequal stress distribution around the septal apex. Preoperative detection of these anatomical variations helps surgeons prepare for the restrictions that might occur [10].

In contrast to previous studies that focused on the evaluation of 2D slices of CBCT scans, the current study based on 3D modules indicates that maxillary sinus septa have different and complicated pathways and orientations other than following the conventional classification system. The spatial distribution and orientation of the septa had an appearance like that of a mountain landscape with many peaks and valleys, irrespective of being complete or incomplete septa or just projections in the maxillary sinus wall, and the septa included continuous changes of direction within the same orientation [19].

However, analysis of the distribution of the septa within the sinus along the alveolar ridge revealed differences in the prevalence of septa in certain tooth positions. The prevalence was highest on the left side in relation to the upper left first molar (tooth #26), where it occurred in 31.43% of cases, while the prevalence on the right side was highest above the upper right second molar (tooth #17), where it occurred in 33.33% of cases. Also, septa were found on the right side in 279 out of 360 patients (77.5%) and on the left side in 289 out of 360 patients (80.27%). This prevalence is substantially higher than the 29.7% reported by Yildirim et al., [13] a discrepancy attributable to fundamental differences in imaging modalities and diagnostic criteria. While conventional CBCT studies rely on sequential 2D axial and coronal slices where slice-interval shifting may fail to identify minor anatomical projections, the present study employed a 3D volumetric de-roofing and lateral resection protocol. This strategy provided a direct, endoscopic-like view of the internal antral floor, enabling the identification of subtle incomplete septa and small bony projections that are frequently overlooked in standard 2D slice-based assessments [13].

Three-dimensional volumetric analysis of the maxillary CBCT represents an increasingly important diagnostic tool in contemporary oral and maxillofacial surgery practice, particularly in cases of sinus floor elevation and dental implant placement. Exact three-dimensional estimation of antral volume provides vital prognostic information regarding the pattern of pneumatization and remaining bone height after extraction, thus allowing better appreciation of the sinus volume, which aids in estimating the required spatial planning for the bone grafting procedure in order to avoid the possibility of excessive bone grafts and inadequate sinus floor elevation. Furthermore, three-dimensional volumetric evaluation plays a significant role in determining the dynamics of the Schneiderian membrane; antral volumes with extensive pneumatization are frequently accompanied by extremely thin mucous linings of the sinuses, hence raising the probability of intraoperative membrane perforation. From the physiological point of view, the measurement of air-filled antrum volume makes it possible to estimate postoperative thickening of the mucous membrane, accumulation of fluid in the cavity, and mucociliary clearance, which is directly connected with the sinus ventilation through the primary ostium [20,21,22].

Quantitative volumetric analysis showed considerable variability in the total maxillary sinus volumes, with male patients having larger mean antral volumes than females. The observed differences in the sizes of sinuses among the sexes may be due to the larger overall size of craniofacial skeleton, increased height of visceral cranium, and powerful masticatory muscle forces in males [3,4].

The semi-automatic active contour segmentation technique (i.e., the “snake” algorithm) used within ITK-SNAP allowed precise measurement of the complicated geometry of the maxillary antrum, thus solving the problem of the inaccurate and distorted estimates obtained by two-dimensional linear estimations. From the reconstructive point of view, the availability of accurate volumetric measurements is particularly useful. It allows surgeons to calculate the precise amount of biomaterial needed for the augmentation procedure and thus avoid under-filling or graft over-packaging.

In addition, volumetric measurements indicated that the average volume of the left-sided maxillary sinus was 14,948.6 mm^3^ while the right-side volume was 13,937.6 mm^3^. Comparing these measurements to values from a Sri Lankan population confirmed similar patterns, but the latter had slightly bigger volumetric dimensions, with the right-sided sinus measuring 14,770 mm^3^ and the left-side measuring 14,740 mm^3^ [23].

Ostial vertical height is one of the essential elements determining antral homeostasis and fluid dynamics. The ostium is the only anatomical opening for mucociliary clearance into the middle nasal meatus [24] and is situated on the upper part of the medial wall of the maxillary sinus. Aggressive vertical sinus floor augmentation poses considerable risks in terms of damaging the ostium in sinuses with a low vertical ostial height or mucosal thickening.

In cases of excessive vertical packing of the graft or upward movement of graft-induced edema following surgery, the functional ostium is compromised with regard to its size, being either narrowed or entirely occluded. Obstruction of the ostium leads to failure of normal mucociliary clearance, resulting in the accumulation of fluid, development of secondary acute rhinosinusitis, and possible infection of the bone graft [24,25]. Thus, measuring ostium height on multi-planar CBCT images can give an important information on the existence of a “safe vertical boundary” which needs to be observed during graft packing.

## 5. Surgical Implications for Sinus Floor Elevation and Risk Mitigation

With regard to the anatomic and imaging features gained through the current CBCT investigation, the following clinical considerations should be made by clinicians when conducting sinus floor augmentation:

1. Pre-surgical Imaging Evaluation: In cases where there are thick mucosa > 2.0 mm together with septa, the imaging features discussed above should be taken into account before surgery. If such abnormalities are present, imaging correlation with clinical/ENT examination is recommended before any surgical interventions [26].
2. Alternative Access Window Design: Where middle septa exist, there is a possibility of using a the single rigid rectangular window for surgery, but a bipartite (double-window) design.
3. Ultrasound Piezo Surgical Application: Since there is a possible injury to the Schneiderian membrane at the sharp edge of the septum, ultrasound piezoelectric devices can be preferred to conventional rotating burs for osteotomy.

## 6. Conclusions

This study demonstrates that the integration of semi-automatic volumetric segmentation with ITK-SNAP in combination with multiple planar anatomical localization by BlueSky Bio offers a reliable diagnostic tool for assessing the variation of the maxillary sinus. In particular, this study found a high rate of occurrence of maxillary sinus septa (77.5–80.27%), and the specific distribution of the structure was characterized by localization in the molar areas (teeth #17 and #26). Maxillary sinus septa have common anatomical variations that show a statistically significant association with mucosal thickening and pathological changes. While these findings do not imply a direct cause-and-effect relationship between septa and sinus disease, the high prevalence of mucosal alterations in septated sinuses highlights the clinical importance of detailed 3D anatomical mapping. Clinicians should carefully evaluate septal morphology and associated mucosal conditions during presurgical planning for sinus floor elevation and endodontic-surgical procedures to minimize complications.

## 7. Limitations and Future Directions

As the sample used for this study was based on a retrospective dental hospital database as opposed to a population sample, any results generated are susceptible to potential biases such as those related to selection or referral.

Diagnosis of lesions on the mucosa and antrum was made by radiographic assessment of CBCT images only. No clinical examinations or pathological confirmation were performed during this study.

The retrospective nature of this study made comparison between clinical manifestations of pathology and histology of sinus biopsies impossible. It is expected that future prospective clinical studies including comparison of preoperative volumetric CBCT assessments and postoperative grafts’ stability as well as histomorphometry of bone maturation will confirm the current study results.

While each maxillary sinus was analyzed as an independent anatomical unit due to side-specific local factors (e.g., localized odontogenic pathologies, asymmetric septal growth, and independent local microenvironments), the bilateral nature of the data (720 sinuses from 360 patients) inherently introduces a potential intra-subject correlation. Although adjusted statistical modeling (GEE and mixed models) confirmed the persistence of statistically significant associations, future prospective studies evaluating side-specific dental and local inflammatory markers are encouraged in order to further elucidate intra-patient vs. inter-patient variances.

Although this study demonstrates the parameters to be independent clinical predictors, further studies should explore potential correlations between them, particularly with regard to the interaction between septal anatomy, ostium height, and mucosal health. The study of these inter-relationships in larger, prospective cohorts may aid in the development of predictive surgical models or AI-powered diagnostic tools to improve pre-surgical augmentation protocols.

## Figures and Tables

**Table 1:** Prevalence and Anatomical Distribution of Maxillary Septa on the Right Side (*n* = 279).

Septa Type	Orientation/Direction	Anatomical Base/Location	Percentage (%)	Number of Cases (*n*)
Complete Septa	Mediolateral	Floor of the Sinus	13.62%	38
Incomplete Septa	Posteroanterior	Sinus Floor	9.32%	26
	Posteroanterior	Medial Wall	9.32%	26
	Posteroanterior	Lateral Wall	9.32%	26
	Superior–inferior	Anterior Wall	9.32%	26
	Superior–inferior	Lateral Wall	9.32%	26
	Mediolateral	Sinus Floor	19.71%	55
	Small Projection	Anterior Wall	20.07%	56
Total			100%	279

**Table 2:** Prevalence and Anatomical Distribution of Maxillary Septa on the Left Side.

Septa Type	Orientation/Direction	Anatomical Base/Location	Percentage (%)	Number of Cases (*n*)
**Complete Septa**	Posterolateral	Sinus Floor	5.88%	17
	Mediolateral	Sinus Floor	1.03%	3
**Incomplete Septa**	Posterolateral	Lateral Wall	31.48%	91
	Superior–inferior	Anterior Wall	19.72%	57
	Small Projection	Sinus Floor	31.48%	91
	Small Projection	Anterior Wall	10.38%	30
**Total**			100%	289

**Table 3:** Morphometric analysis of the vertical height of the maxillary sinus ostium according to side and sex.

Anatomical Location/Group	Sample Group	Mean ± SD (mm)	*p*-Value
**Overall Comparison**	Left Side (Total)	26.20 ± 5.10	0.501
	Right Side (Total)	25.90 ± 5.45	
**Left Side**	Males	28.65 ± 3.28	0.007 *
	Females	23.79 ± 5.16	
**Right Side**	Males	27.54 ± 5.76	0.113
	Females	24.32 ± 4.98	

* statistical significant.

**Table 4:** Comparative Analysis of Maxillary Sinus Findings Between the Left and Right Sides.

Condition	Left Side (*n*, %)	Right Side (*n*, %)	*p*-Value
**Membrane Thickening**	101 (28.05%)	63 (17.50%)	0.0012 *
**Thickening at the Roof**	-	9 (2.5%)	-
**Partial Obstruction**	-	9 (2.5%)	-
**Mucocele**	63 (17.50%)	63 (17.50%)	1.0000

* Statistically Significant.

## Data Availability

The data presented in this study are available on request from the corresponding author.

## Abbreviations

The following abbreviations are used in this manuscript:

CBCT	Cone-beam computed tomography
SD	Standard deviation
2D	Two dimensional
3D	Three dimensional
